# Ambient air pollution and mortality: The role of socioeconomic conditions

**DOI:** 10.1097/EE9.0000000000000297

**Published:** 2024-03-07

**Authors:** Felipe Parra do Nascimento, Nelson Gouveia

**Affiliations:** a School of Medicine, University of São Paulo, São Paulo, Brazil

**Keywords:** air pollution, mortality, nitrogen dioxide, ozone, particulate matter, socioeconomic status

## Abstract

**Background:**

There is a vast body of literature covering the association between air pollution exposure and nonaccidental mortality. However, the role of socioeconomic status (SES) in this relationship is still not fully understood.

**Objectives:**

We investigated if individual and contextual SES modified the relationship between short-term exposure to ozone (O_3_), nitrogen dioxide (NO_2_), and particulate matter with aerodynamic diameter <10 µm (PM_10_) on cardiovascular, respiratory, and all nonaccidental mortality.

**Methods:**

We conducted a time-stratified case-crossover study. Analyses were based on information on 280,685 deaths from 2011 to 2015 in the city of São Paulo. Education was used as an individual SES, and information on the district of residence was used to build a contextual SES. Exposure to PM_10_, NO_2,_ and O_3_ was accessed from monitoring stations and linked to each case based on the date of death. Conditional logistic regression models were used to estimate the effects of air pollutants, and interaction terms were added to access the effect modification of SES.

**Results:**

Individuals with lower education had an increased chance of dying for all nonaccidental outcomes (1.54% [0.91%, 2.14%]) associated with exposure to PM_10_. Individuals living in lower SES areas had an increased chance of dying for nonaccidental (0.52% [0.16%, 0.88%]), cardiovascular (1.17% [0.88%, 1.46%]), and respiratory (1.70% [0.47%, 2.93%]) causes owing to NO_2_ exposure.

**Conclusion:**

Exposure to air pollutants increases the chance of dying by nonaccidental, cardiovascular, and respiratory causes. Lower educational levels and living on lower contextual SES increased the risk of mortality associated with air pollution exposure.

**What the study adds**

This study shows the effect of modification of both individual and area-level socioeconomic status on the air pollution-mortality relationship in São Paulo City, Brazil. It explores the impact of PM_10_, O_3,_ and NO_2_ short-term exposure on nonaccidental, cardiovascular, and respiratory mortality.

## Introduction

Air pollution has been identified as a serious public health problem since the first half of the 20th century. The main sources of emission in urban areas include industrial processes and the burning of fossil fuels.^1^ Age groups most affected are children^2,3^ and elderly.^4,5^ Various studies, systematic reviews, and meta-analyses documented associations between air pollutants such as nitrogen dioxide (NO_2_), particulate matter with aerodynamic diameter <10 µm (PM_10_), and ozone (O_3_) and adverse health outcomes.^5–11^

Studies have evaluated how air pollution exposure and socioeconomic status (SES) correlate with mortality and if SES modifies the effects of air pollution on the population. The main hypotheses are that either poorer individuals are more exposed to air pollution directly in their jobs or living near industrial sources (environmental injustice) or that individuals with lower SES become more vulnerable to the harmful effects of air pollution because of limited access to healthy food and water, limited access to health care services, and worse lifestyle habits such as smoking and drinking.^6,12,13^

Some studies have already reported that both individual and area-level SES modify the effects of air pollution on the health of the population. A case-crossover study conducted in São Paulo, Brazil, reported that the effect of short-term exposure to NO_2_, SO_2,_ and CO on nonaccidental mortality was modified by individual-level SES (education).^7^ A similar finding was reported in a time-series study in China, where low educational levels modified the short-term effect of PM_10_ on all-cause mortality.^14^ An ecological study in Wales, UK, reported a higher chance of dying owing to all-cause and respiratory diseases owing to PM_10_, PM_2.5,_ and NO_2_ short-term exposure on low area-level SES,^15^ similar to a case-crossover study in Paris, France, that reported a higher risk of all-cause mortality owing to both short- and long-term exposure to NO_2_ among individuals living in most deprived census blocks.^6^ In a cohort conducted in the United States, it was reported that the association between long-term exposure to PM_2.5_ and all-cause mortality was modified by both individual and area-level SES, with the lowest SES levels being the most vulnerable.^16^ On the other hand, some studies have reported associations between air pollution and adverse health effects but with no SES modification,^17–21^ while in others the effect was higher among those with higher SES.^22,23^

Given the contradictory results observed in the literature and the scarcity of studies in Latin America that jointly address individual and area-level SES, the present study aims to contribute to the body of knowledge in this area by examining the role of individual and contextual SES as effect modifiers in the association between short-term exposure to air pollution and mortality in Brazil. Our study takes place in São Paulo, Brazil, a city with large socioeconomic disparities and high levels of air pollution. São Paulo has a lot of social inequality, having a Gini coefficient of 0.62 in 2010.^24^ Based on previous evidence, we have chosen PM_10_, O_3,_ and NO_2_ as the air pollutants to be analyzed in relation to mortality by all nonaccidental causes, respiratory diseases, and cardiovascular diseases.

## Methods

### Study area

This study was carried out in the municipality of São Paulo, Brazil’s largest city, with a population of over 12 million people in 2016 and a demographic density of 7960 inhabitants/km².^24^

### Mortality data

Daily deaths were collected by Programa de Aprimoramento das Informações de Mortalidade (PRO-AIM), São Paulo’s municipal health secretary program, from 1 January 2011 to 31 December 2015. We obtained information from death certificates on individual age, sex, date of death, education, district of residence, and main cause of death according to international classification of diseases. The outcomes analyzed were deaths by all nonaccidental causes (A00–T98, Z00–Z99), owing to respiratory (J00–J98) and cardiovascular diseases (I00–I99). We excluded individuals less than 30 years old for all analyses.

### Air pollution data

Data for air pollution were extracted directly from the Companhia Ambiental do Estado de São Paulo Monitoring Network System from 1 January 2010 to 31 December 2015. We included the year 2010 because deaths that happened in early 2011 had air pollution exposure in 2010. We obtained the daily mean value for PM_10_ and NO_2_ and the highest 8-hour moving average for O_3_ in µg/m³ for each monitoring station. We considered using PM_2.5_ in our analysis but the data was available only for 5 monitoring stations and had more than 20% missing data, which would compromise the results. Daily air pollutant measures were considered valid when at least 16 of the 24-hourly measurements had no errors. PM_10_ was measured at 13 monitoring stations while O_3_ at 12 and NO_2_ at 9. Because the correlation between monitoring stations for each pollutant was very high (see Supplementary Material, Tables S1–S3, http://links.lww.com/EE/A266), when data were available for more than one monitoring station, the values were averaged to create a citywide pollutant concentration.

### Meteorological data

Daily average temperature and relative humidity were obtained from the Instituto de Astronomia, Geofísica, e Ciências Atmosféricas da Universidade de São Paulo from 1 January 2010 to 31 December 2015 period.

### Socioeconomic status

We used education attainment as an individual SES indicator which was divided into three categories: 0–8 (“low”), 9–11 (“middle”), and 12 or more years of study (“high”). Details of the approach to calculate area-level SES can be found elsewhere.^25^ In summary, we created a socioeconomic index using information from the 2010 Census for each of the 96 districts of São Paulo. The index was constructed by applying principal components analysis with varimax rotation to the following variables: mean income; literacy rate; one-person housing; education (mean years of education for head of household); and housing conditions (proportion of residences not connected to sewage network). The 32 districts with the highest index were allocated in the “high” category, the following 32 into the “middle” category, and the lowest 32 into the “low” category. Individuals were then allocated to the SES of the district of their residence.

### Study design and statistical analysis

To study the effects of air pollution in São Paulo, we conducted a time-stratified case-crossover design where each case acts as its own control. We used a time-stratified referent selection in which time was divided into fixed strata and days in each stratum were considered as referents. The control days were matched on the same rounded daily temperature in the same month and year when a case (death) happened, excluding 7 days before the event to eliminate the 6-day autocorrelation between all observations used in the analysis as well as seasonality.^26^

First, we estimated the risk of mortality for nonaccidental, cardiovascular, and respiratory diseases owing to exposure to PM_10_, NO_2_, and O_3_ through a multiple conditional logistic regression adjusting by age, sex, and relative humidity. Pollutants entered the models as continuous variables on single-pollutant models. Finally, we added a multiplicative interaction term, with high SES (both individual and area-level) as the reference category, adjusting for age, sex, and relative humidity for the same outcomes of the previous model. All results are reported as percent change in the odds ratios ([OR − 1]*100%) for an increase of 10 µg/m³ of the pollutant in the case period in relation to the control period, followed by the 95% confidence interval (95% CI) for each estimate. All statistical analyses were performed using Stata version 15.0.

## Results

From 1 January 2011 to 31 December 2015, we had a total of 311,574 deaths for nonaccidental causes in São Paulo municipality for individuals over 30 years of age. We excluded individuals who had no information about educational level in the death certificates (30,889 or 9.9%) from all analyses. Because all other studied variables had no missing data, our final study population was 280,685 individuals. Subjects were mostly between 75 and 109 years (47.1%), between 0 and 8 years of study (70.9%), and lived in low area-SES districts (38.4%). Similar proportions were observed when broken down by cardiovascular and respiratory outcomes (Table 1). Mean age for all nonaccidental deaths was 71.1 years (SD = 15.3), similar to cardiovascular and respiratory outcomes. Women were on average 5.7 years older than men and less educated (12.6% of men had 12 or more years of study versus 7.9% among women). There were more females than males that lived in high SES districts (29.9 versus 25.7%). Mean age among the low-education group was 72.8 years, while in the high-education group it was 68.7 years. Mean age of individuals living in low-SES districts was 66.7 years while in high-SES districts was 76.4 years (data not shown).

**Table 1.** Descriptive statistics for the study population, São Paulo, 2011–2015

	Nonaccidental N (%)	Cardiovascular N (%)	Respiratory N (%)
Total	280,685	102,032	40,973
Age group			
30–64	91,859 (32.7)	31,038 (30.4)	8,780 (21.5)
65–74	56,701 (20.2)	21,388 (21.0)	7,254 (17.8)
75–109	132,125 (47.1)	49,606 (48.6)	24,759 (60.7)
Sex			
Male	140,594 (50.1)	51,200 (50.2)	20,716 (50.8)
Female	140,091 (49.9)	50,832 (49.8)	20,077 (49.2)
Years of study			
0–8	199,086 (70.9)	75,027 (73.5)	31,032 (76.1)
9–11	52,755 (18.8)	18,503 (18.1)	6,408 (15.7)
12+	28,844 (10.3)	8,502 (8.4)	3,353 (8.2)
Area-SES			
Low	107,737 (38.4)	41,994 (41.2)	15,137 (37.1)
Medium	94,851 (33.8)	34,607 (33.9)	13,810 (33.9)
High	78,097 (27.8)	25,431 (24.9)	11,846 (29.0)

SES, socioeconomic status.

During the study period, the daily mean level of PM_10_ was 38.7 µg/m³ (SD = 16.0 µg/m³), NO_2_ was 78.9 µg/m³ (SD = 33.5 µg/m³), and the highest daily 8-hour moving average of O_3_ was 69.4 µg/m³ (SD = 32.0 µg/m³). The mean daily temperature was around 20ºC and the relative humidity was around 80% (Table 2). The correlation between pollutants was high for PM_10_ and NO_2_ (Pearson r = 0.750) but not so high for O_3_ (Pearson r = 0.339). O_3_ was correlated with temperature (Pearson r =0.593) and inversely correlated with relative humidity (Pearson r = −0.600) (full correlation matrix available on Supplementary Material, Table S4, http://links.lww.com/EE/A266).

**Table 2.** Air pollutants concentrations and meteorological measures, São Paulo, 2011–2015

	Mean	SD	Min	25th	Median	75th	Max
O_3_ (µg/m³)	69.4	32.0	3.7	45.0	65.3	90.7	187.0
PM_10_ (µg/m³)	38.7	16.0	9.5	27.0	35.7	46.7	110.7
NO_2_ (µg/m³)	78.9	33.5	11.0	54.0	74.0	100.0	209.0
Temperature (°C)	19.7	3.3	7.3	17.5	19.8	22.2	28.0
Relative humidity (%)	79.8	8.9	41.2	74.9	80.8	86.0	96.9

NO_2_, nitrogen dioxide; O_3_, ozone; PM_10_, particulate matter with aerodynamic diameter <10 µm.

In models adjusted for age, sex, and relative humidity, a 10 µg/m³ increase in levels of O_3_ led to a 0.52% (95% CI = 0.46%, 0.54%) increase in all nonaccidental mortality, 0.49% increase (95% CI = 0.40%, 0.57%) in cardiovascular and 0.53% increase (95% CI = 0.40%, 0.66%) in respiratory mortality. An increase of 10 µg/m³ of NO_2_ exposure was associated with a 1.13% (95% CI = 1.12%, 1.15%) increase in nonaccidental mortality, 1.11% (95% CI = 1.05%, 1.17%) increase in cardiovascular mortality and 1.12% (95% CI = 1.10, 1.15%) increase in respiratory mortality. For PM_10_, an increase of 10 µg/m³ in the exposure was associated with increases in mortality, but the estimates were not statistically significant (Table 3).

**Table 3.** Percentage change (95% confidence interval) of mortality associated with an increase of 10 µg/m³ in air pollutant concentration^a^

	O_3_	PM_10_	NO_2_
Nonaccidental	0.52 (0.46, 0.54)	0.38 (−0.18, 0.94)	1.13 (1.12, 1.15)
Cardiovascular	0.49 (0.40, 0.57)	0.06 (−0.86, 0.26)	1.11 (1.05, 1.17)
Respiratory	0.53 (0.40, 0.66)	0.62 (−0.96, 1.28)	1.12 (1.10, 1.15)

a Adjusted by age, sex, and relative humidity.

NO_2_, nitrogen dioxide; O_3_, ozone; PM_10_, particulate matter with aerodynamic diameter <10 µm.

In models that tested for effect modification of individual and area-level SES (Tables 4–6), we observed that the effect of O_3_ on mortality did not seem to be modified by either individual or area-level SES (Table 4).

**Table 4.** Percentage change (95% confidence intervals) of interaction between O_3_ exposure and individual and area-level SES, for an increase of 10 μg/m

	Nonaccidental	Cardiovascular	Respiratory
Education			
12+ years	0	0	0
9–11 years	0.57 (0.37, 0.77)	0.87 (−0.84, 1.90)	0.58 (−0.07, 1.13)
0–8 years	0.03 (−0.14, 0.17)	0.57 (−0.64, 1.78)	0.09 (−0.59, 0.77)
Area-SES			
High	0	0	0
Mid	0.36 (−0.04, 0.72)	0.35 (−0.26, 0.96)	0.16 (−0.51, 0.83)
Low	1.91 (−0.08, 3.00)	0.25 (−0.81, 0.69)	0.45 (−0.14, 1.04)

SES, socioeconomic status.

On the other hand, for PM_10_, we found that individual SES modified the effect of the air pollution-nonaccidental mortality association. Individuals who had 0–8 years of study had an additional 1.54% (95% CI = 0.91%, 2.14%) chance of dying for nonexternal causes, while individuals with 9–11 years of study had an additional 0.97% (95% CI = 0.15%, 1.79%) chance of dying for the same outcome, in a dose-response relationship (Table 5).

**Table 5.** Percentage change (95% confidence intervals) of interaction between PM_10_ exposure and individual and area-level SES, for an increase of 10 μg/m³

	Nonaccidental	Cardiovascular	Respiratory
Education			
12+ years	0	0	0
9–11 years	0.97 (0.15, 1.79)	0.13 (−0.83, 0.43)	0.95 (−0.14, 2.14)
0–8 years	1.54 (0.91, 2.14)	0.26 (0.03, 0.29)	−0.87 (−2.36, 0.67)
Area-SES			
High	0	0	0
Mid	0.09 (−0.37, 0.55)	−0.07 (−0.81, 0.67)	1.48 (0.08, 2.88)
Low	0.01 (−0.78, 0.80)	−0.36 (−1.64, 0.98)	1.15 (−0.09, 2.39)

SES, socioeconomic status.

We also found a dose-response relationship of area-SES modifying the effect of increased exposure to NO_2_ and nonaccidental, cardiovascular, and respiratory outcomes (Table 6). Individuals living in districts with low area SES had an additional chance of dying for nonaccidental (0.52%), cardiovascular (1.17%), and respiratory (1.70%) causes.

**Table 6.** Percentage change (95% confidence intervals) of interaction between NO_2_ exposure and individual and area-level SES, for an increase of 10 μg/m³

	Nonaccidental	Cardiovascular	Respiratory
Education			
12+ years	0	0	0
9–11 years	−0.05 (−0.90, 0.85)	−0.23 (−0.98, 0.52)	0.15 (−0.21, 0.51)
0–8 years	−0.07 (−0.92, 0.78)	−0.12 (−1.13, 0.89)	0.08 (−0.52, 0.68)
Area-SES			
High	0	0	0
Mid	0.37 (0.18, 0.56)	0.57 (0.18, 0.96)	0.56 (0.01, 1.11)
Low	0.52 (0.16, 0.88)	1.17 (0.88, 1.46)	1.70 (0.47, 2.93)

SES, socioeconomic status.

## Discussion

This study found some evidence that individual SES may modify the effect of short-term exposure to PM_10_ on nonaccidental mortality, and area-level SES modifies the effect of NO_2_ on daily mortality from nonaccidental, cardiovascular, and respiratory outcomes. On the other hand, we did not observe the effect modification of individual or area-level SES in the association between O_3_ and mortality.

Our findings agree with previous results reported in different places. A cohort study in Canada reported that individuals with education lower than high school had a higher risk of dying from nonaccidental causes associated with long-term exposure to PM_2.5_ than those with more years of study (hazard ratio [HR] = 1.060; 95% CI = 1.042, 1.079).^27^ A time-series study in the United States reported a higher risk of death for cardiorespiratory causes in individuals with 0–12 years of schooling than those with more than 12 years associated with an increase of 10 µg/m³ in both short- and long-term PM_2.5_ exposure (OR = 1.90; 95% CI = 1.60, 2.10 in lower education versus OR = 1.40; 95% CI = 1.20, 1.60 in higher education).^28^

Effect modification by SES in the association between short-term NO_2_ exposure and all-cause mortality has been documented in a case-crossover study in France, where individuals living in most deprived census blocks had statistically higher risks of dying compared with mid and less deprived blocks (excess risk % = 3.14; 95% CI = 1.41, 4.90).^6^

Other studies reported effect modification in the opposite direction, such as the Chinese cohort that found a higher risk ratio for cardiovascular mortality associated with an increase of 10 µg/m³ in long-term PM_10_ exposure in the high education group (risk ratio = 1.52; 95% CI = 1.41, 1.65 in relation to risk ratio = 1.19; 95% CI = 1.15, 1.22 in the low education category).^23^ On the other hand, many studies that aimed to find a modification of the effect of air pollutants on mortality by individual SES reported no evidence of this.^19–21,29,30^ Comparison between estimates of health impacts from other studies may not be completely viable owing to differences in air pollution mixture, health care systems, climate, and definition of SES.^7^

The plausibility of individuals with lower SES being more susceptible to air pollutants has been suggested previously and it was proposed that low SES may indicate worse housing conditions, which could also indicate higher indoor pollution, less access to healthy food and clean water, and unhealthy lifestyle such as smoking, leading to a predisposition to obesity, diabetes, and other comorbidities.^31^

Our study had some strengths, such as the use of both individual data collected from death certificates and contextual information based on an index constructed from principal components analysis that was able to socioeconomically divide the entire population of the municipality. Our study had also some limitations. It was not possible to measure each individual exposure to pollution because we could not access occupational exposure or smoking habits. Our area-level SES index did not take into account the geographic extension or the population size in each area, which could have led to a misclassification in SES.

## Conclusions

In conclusion, we report further evidence for the association between O_3_ and NO_2_ short-term exposure and nonaccidental, cardiovascular, and respiratory mortality. We could not find any effect modification of SES in the association between short-term exposure to O_3_ and mortality but we were able to detect effect modification by individual-level SES in the association between short-term exposure to PM_10_ and nonaccidental mortality, where individuals with lower schooling had increased chance of dying. Area-level SES modified the association between short-term exposure to NO_2_ and mortality by nonaccidental, cardiovascular, and respiratory outcomes, where individuals living on lower area-level SES had an increased chance of dying.

## Conflicts of interest statement

The authors declare that they have no conflicts of interest with regard to the content of this report.

## Supplementary Material



## References

[1] Braga ALF Pereira LAA Procópio M André PA Saldiva PHN. Associação entre poluição atmosférica e doenças respiratórias e cardiovasculares na cidade de Itabira, Minas Gerais, Brasil. Cad Saude Publica. 2007;23:5570–5578. 10.1590/s0102-311x2007001600017 18038038

[2] Ghosh JKC Heck JE Cockburn M Su J Jerrett M Ritz B. Prenatal exposure to traffic-related air pollution and risk of early childhood cancers. Am J Epidemiol. 2013;178:1233–1239. 23989198 10.1093/aje/kwt129 PMC3792733

[3] Carbajal-Arroyo L Miranda-Soberanis V Medina Ramón M. Effect of PM_10_ and O_3_ on infant mortality among residents in the Mexico City Metropolitan Area: a case-crossover analysis, 1997-2005. J Epidemiol Community Health. 2011;65:715–721. 20724286 10.1136/jech.2009.101212

[4] Cakmak S Dales RE Rubio MA Vidal CB. The risk of dying on days of higher air pollution among the socially disadvantaged elderly. Environ Res. 2011;111:388–393. 21256481 10.1016/j.envres.2011.01.003

[5] Yang Y Tang R Qiu H. Long term exposure to air pollution and mortality in an elderly cohort in Hong Kong. Environ Int. 2018;117:99–106. 29730535 10.1016/j.envint.2018.04.034

[6] Deguen S Petit C Delbarre A. Neighbourhood characteristics and long-term air pollution levels modify the association between the short-term nitrogen dioxide concentrations and all-cause mortality in Paris. PLoS One. 2015;10:14–26. 10.1371/journal.pone.0131463 PMC4510557 26197409

[7] Bravo MA Son J Freitas CU Gouveia N Bell ML. Air pollution and mortality in São Paulo, Brazil: effects of multiple pollutants and analysis of susceptible populations. J Expo Sci Environ Epidemiol. 2016;26:150–161. 25586330 10.1038/jes.2014.90

[8] Orellano P Reynoso J Quaranta N Bardach A Ciapponi A. Short-term exposure to particulate matter (PM_10_ and PM_2.5_), nitrogen dioxide (NO_2_) and ozone (O_3_) and all-cause and cause-specific mortality: systematic review and meta-analysis. Environ Int. 2020;142:105876. 10.1016/j.envint.2020.105876 32590284

[9] Song Q Christiani DC Wang X. The Global contribution of outdoor air pollution to the incidence, prevalence, mortality and hospital admissions for chronic obstructive pulmonary disease: a systematic review and meta-analysis. Int J Environ Res Public Health. 2014;11:11822–11832. 25405599 10.3390/ijerph111111822 PMC4245645

[10] Wang M Li H Huang S. Short-term exposure to nitrogen dioxide and mortality: a systematic review and meta-analysis. Environ Res. 2021;202:111766–111782. 34331919 10.1016/j.envres.2021.111766 PMC8578359

[11] Zhao L Liang HR Chen FY Chen Z Guam WJ Li JH. Association between air pollution and cardiovascular mortality in China: a systematic review and meta-analysis. Oncotarget. 2017;8:66438–66448. 29029525 10.18632/oncotarget.20090 PMC5630425

[12] Wong CM Ou CQ Chan KP. The effects of air pollution on mortality in socially deprived urban areas in Hong Kong, China. Environ Health Perspect. 2008;116:1189–1194. 18795162 10.1289/ehp.10850 PMC2535621

[13] Qiu H Tian L Ho KF Pun VC Wang X Yu ITS. Air pollution and mortality: effect modification by personal characteristics and specific cause of death in a case-only study. Environ Pollut. 2015;199:192–197. 25679980 10.1016/j.envpol.2015.02.002

[14] Chen R Kan H Chen B; CAPES Collaborative Group. Association of particulate air pollution with daily mortality: the China air pollution and health effects study. Am J Epidemiol. 2012;175:1173–1181. 22510278 10.1093/aje/kwr425

[15] Brunt H Barnes J Jones SJ Longhurst JWS Scally G Hayes E. Air pollution, deprivation and health: understanding relationships to add value to local air quality management policy and practice in Wales, UK. J Public Health (Oxf). 2016;39:485–497. 10.1093/pubmed/fdw084 27613763

[16] Wang Y Shi L Lee M. Long-term exposure to PM2.5 and mortality among older adults in the Southeastern US. Epidemiology. 2017;28:207–214. 28005571 10.1097/EDE.0000000000000614 PMC5285321

[17] Chi GC Hajat A Bird CE. Individual and neighborhood socioeconomic status and the association between air pollution and cardiovascular disease. Environ Health Perspect. 2016;124:1840–1847. 27138533 10.1289/EHP199 PMC5132637

[18] Dong GH Zhang P Sun B. Long-term exposure to ambient air pollution and respiratory disease mortality in Shenyang, China: a 12-year population-based retrospective cohort study. Respiration. 2012;84:360–368. 22116521 10.1159/000332930

[19] Jerrett M Burnett RT Pope CA 3rd. Long-term ozone exposure and mortality. N Engl J Med. 2009;360:1085–1095. 19279340 10.1056/NEJMoa0803894 PMC4105969

[20] O’Neill MS Bell ML Ranjit N. Air pollution and mortality in Latin America: the role of education. Epidemiology. 2008;19:810–819. 18633327 10.1097/EDE.0b013e3181816528

[21] Ren C Melly S Schwartz J. Modifiers of short-term effects of ozone on mortality in eastern Massachusetts: a case-crossover analysis at individual level. Environ Health. 2010;9:3. 20092648 10.1186/1476-069X-9-3 PMC2825215

[22] Schifano P Lallo A Asta F De Sario M Davoli M Michelozzi P. Effect of ambient temperature and air pollutants on the risk of preterm birth, Rome 2001-2010. Environ Int. 2013;61:77–87. 24103349 10.1016/j.envint.2013.09.005

[23] Zhang LW Chen X Xue XD. Long-term exposure to high particulate matter pollution and cardiovascular mortality: a 12-year cohort study in four cities in northern China. Environ Int. 2014;62:41–47. 24161381 10.1016/j.envint.2013.09.012

[24] Instituto Brasileiro de Geografia e Estatística. Rio de Janeiro: IBGE – Instituto Brasileiro de Geografia e Estatística; c2017 [cited 2018 May 10]. São Paulo [municipality]: panorama. Available from: https://cidades.ibge.gov.br/brasil/sp/sao-paulo/panorama

[25] Nascimento FP Almeida MF Gouveia N. Individual and contextual socioeconomic status as effect modifier in the air pollution-birth outcome association. Sci Total Environ. 2022;803:149790. 34481165 10.1016/j.scitotenv.2021.149790

[26] Levy D Lumley T Sheppard L Kaufman J Checkoway H. Referent selection in case-crossover analysis of acute health effects of air pollution. Epidemiology. 2001;12:186–192. 11246579 10.1097/00001648-200103000-00010

[27] Pinault LL Weichenthal S Crouse DL. Associations between fine particulate matter and mortality in the 2001 Canadian Census Health and Environment Cohort. Environ Res. 2017;159:406–415. 28850858 10.1016/j.envres.2017.08.037

[28] Kloog I Ridgway B Koutrakis P Coull BA Schwartz JD. Long- and short-term exposure to PM_2.5_ and mortality: using novel exposure models. Epidemiology. 2013;24:555–561. 23676266 10.1097/EDE.0b013e318294beaa PMC4372644

[29] Ou CQ Hedley AJ Chung RY. Socioeconomic disparities in air pollution-associated mortality. Environ Res. 2008;107:237–244. 18396271 10.1016/j.envres.2008.02.002

[30] Faustini A Stafoggia M Renzi M. Does chronic exposure to high levels of nitrogen dioxide exacerbate the short-term effects of airborne particles? Occup Environ Med. 2016;73:772–778. 27503102 10.1136/oemed-2016-103666

[31] O’Neill MS Jerrett M Kawachi I; Workshop on Air Pollution and Socioeconomic Conditions. Health, wealth, and air pollution: advancing theory and methods. Environ Health Perspect. 2003;111:1861–1870. 14644658 10.1289/ehp.6334 PMC1241758

