# Supplementary material for: Ambient air pollution and mortality: The role of socioeconomic conditions
Source: Environ Epidemiol. 2024 Mar 7;8(2):e297. doi: 10.1097/EE9.0000000000000297 (PMC11008627; doi:10.1097/EE9.0000000000000297)
Supplement: Supplementary file 1 [file ee9-8-e297-s001.docx]

Table S1: NO_2_ Correlation between monitoring stations

|  | Pq D. Pedro II | Congonhas | Ibirapuera | Cerqueira Cesar | Ipen | Parelheiros | Pinheiros | Interlagos | Capão Redondo |
| --- | --- | --- | --- | --- | --- | --- | --- | --- | --- |
| Congonhas | 0.752 |  |  |  |  |  |  |  |  |
| Ibirapuera | 0.881 | 0.784 |  |  |  |  |  |  |  |
| Cerqueira Cesar | 0.867 | 0.810 | 0.865 |  |  |  |  |  |  |
| Ipen - USP | 0.839 | 0.731 | 0.830 | 0.837 |  |  |  |  |  |
| Parelheiros | 0.724 | 0.648 | 0.730 | 0.779 | 0.750 |  |  |  |  |
| Pinheiros | 0.823 | 0.783 | 0.831 | 0.866 | 0.829 | 0.746 |  |  |  |
| Interlagos | 0.810 | 0.687 | 0.851 | 0.819 | 0.829 | 0.857 | 0.819 |  |  |
| Capão Redondo | 0.825 | 0.695 | 0.807 | 0.826 | 0.860 | 0.876 | 0.806 | 0.907 |  |
| Marg Tiete | 0.840 | 0.788 | 0.811 | 0.879 | 0.810 | 0.806 | 0.855 | 0.811 | 0.839 |

Table S2: PM_10_ Correlation between monitoring stations

|  | Santana | Pq D. P II | Congonhas | Ibirapuera | Mooca | Cerqueira Cesar | N Sra do Ó | Parelheiros | Santo Amaro | Interlagos | Marg Tiete | Capão Redondo | Pinheiros |
| --- | --- | --- | --- | --- | --- | --- | --- | --- | --- | --- | --- | --- | --- |
| Pq D. P II | 0.823 |  |  |  |  |  |  |  |  |  |  |  |  |
| Congonhas | 0.818 | 0.860 |  |  |  |  |  |  |  |  |  |  |  |
| Ibirapuera | 0.800 | 0.809 | 0.792 |  |  |  |  |  |  |  |  |  |  |
| Mooca | 0.826 | 0.817 | 0.837 | 0.766 |  |  |  |  |  |  |  |  |  |
| Cerqueira Cesar | 0.831 | 0.842 | 0.851 | 0.760 | 0.856 |  |  |  |  |  |  |  |  |
| N Sra do Ó | 0.735 | 0.743 | 0.738 | 0.679 | 0.717 | 0.691 |  |  |  |  |  |  |  |
| Parelheiros | 0.515 | 0.509 | 0.532 | 0.591 | 0.540 | 0.636 | 0.423 |  |  |  |  |  |  |
| Santo Amaro | 0.819 | 0.836 | 0.835 | 0.862 | 0.861 | 0.893 | 0.628 | 0.562 |  |  |  |  |  |
| Interlagos | 0.835 | 0.859 | 0.865 | 0.862 | 0.878 | 0.882 | 0.683 | 0.540 | 0.894 |  |  |  |  |
| Marg Tiete | 0.551 | 0.570 | 0.608 | 0.399 | 0.578 | 0.703 | 0.558 | 0.632 | 0.580 | 0.573 |  |  |  |
| Capão Redondo | 0.773 | 0.803 | 0.781 | 0.876 | 0.825 | 0.858 | 0.792 | 0.572 | 0.844 | 0.886 | 0.621 |  |  |
| Pinheiros | 0.804 | 0.838 | 0.873 | * | 0.832 | 0.889 | 0.812 | 0.593 | 0.846 | 0.862 | 0.740 | 0.834 |  |
| Itaim Paulista | 0.849 | 0.845 | 0.857 | * | 0.885 | 0.899 | 0.850 | 0.567 | 0.837 | 0.830 | 0.693 | 0.802 | 0.845 |

* Was not possible to calculate the correlation

Table S3: O_3_ Correlation between monitoring stations

|  | Santana | Santo Amaro | Pq D P II | Ibirapuera | Mooca | Ipen | N Sra do Ó | Itaquera | Parelheiros | Pinheiros | Interlagos | Itaim Paulista |
| --- | --- | --- | --- | --- | --- | --- | --- | --- | --- | --- | --- | --- |
| Santo Amaro | 0.865 |  |  |  |  |  |  |  |  |  |  |  |
| Pq D P II | 0.897 | 0.883 |  |  |  |  |  |  |  |  |  |  |
| Ibirapuera | 0.867 | 0.909 | 0.893 |  |  |  |  |  |  |  |  |  |
| Mooca | 0.858 | 0.853 | 0.866 | 0.854 |  |  |  |  |  |  |  |  |
| Ipen | 0.885 | 0.936 | 0.897 | 0.902 | 0.836 |  |  |  |  |  |  |  |
| N Sra do Ó | 0.945 | 0.851 | 0.871 | 0.855 | 0.834 | 0.876 |  |  |  |  |  |  |
| Itaquera | 0.854 | 0.830 | 0.811 | 0.836 | 0.824 | 0.823 | 0.843 |  |  |  |  |  |
| Parelheiros | 0.771 | 0.914 | 0.791 | 0.821 | 0.805 | 0.820 | 0.763 | 0.792 |  |  |  |  |
| Pinheiros | 0.780 | 0.881 | 0.850 | 0.876 | 0.801 | 0.882 | 0.783 | 0.763 | 0.744 |  |  |  |
| Interlagos | 0.878 | 0.941 | 0.852 | 0.898 | 0.819 | 0.906 | 0.878 | 0.867 | 0.894 | 0.826 |  |  |
| Itaim Paulista | 0.886 | 0.847 | 0.857 | 0.846 | 0.862 | 0.855 | 0.866 | 0.898 | 0.795 | 0.807 | 0.852 |  |
| Capão Redondo | 0.849 | 0.905 | 0.814 | 0.861 | 0.833 | 0.892 | 0.852 | 0.787 | 0.837 | 0.822 | 0.911 | 0.800 |

Table S4: Correlation Between pollutants and meteorological parameters

|  | O_3_ | PM_10_ | NO_2_ | Temperature |
| --- | --- | --- | --- | --- |
| PM_10_ | 0.339 |  |  |  |
| NO_2_ | 0.422 | 0.750 |  |  |
| Temperature | 0.593 | 0.031 | 0.189 |  |
| Relative humidity | -0.600 | -0.551 | -0.495 | -0.384 |
